# Chemogenetic Suppression of GnRH Neurons during Pubertal Development Can Alter Adult GnRH Neuron Firing Rate and Reproductive Parameters in Female Mice

**DOI:** 10.1523/ENEURO.0223-20.2020

**Published:** 2020-06-17

**Authors:** Eden A. Dulka, R. Anthony DeFazio, Suzanne M. Moenter

**Affiliations:** 1 Department of Molecular and Integrative Physiology, University of Michigan, Ann Arbor, MI 48109; 2 Department of Internal Medicine, University of Michigan, Ann Arbor, MI 48109; 3 Department of Obstetrics and Gynecology, University of Michigan, Ann Arbor, MI 48109

**Keywords:** chemogenetic, development, infertility, neural activity, neuroendocrine

## Abstract

Gonadotropin-releasing hormone (GnRH) neurons control anterior pituitary, and thereby gonadal, function. GnRH neurons are active before outward indicators of puberty appear. Prenatal androgen (PNA) exposure mimics reproductive dysfunction of the common fertility disorder polycystic ovary syndrome (PCOS) and reduces prepubertal GnRH neuron activity. Early neuron activity can play a critical role in establishing circuitry and adult function. We tested the hypothesis that changing prepubertal GnRH neuron activity programs adult GnRH neuron activity and reproduction independent of androgen exposure in female mice. Activating (3Dq) or inhibitory (4Di) designer receptors exclusively activated by designer drugs (DREADDs) were targeted to GnRH neurons using Cre-lox technology. In control studies, the DREADD ligand clozapine n-oxide (CNO) produced the expected changes in GnRH neuron activity *in vitro* and luteinizing hormone (LH) release *in vivo*. CNO was administered to control or PNA mice between two and three weeks of age, when GnRH neuron firing rate is reduced in PNA mice. In controls, reducing prepubertal GnRH neuron activity with 4Di increased adult GnRH neuron firing rate and days in diestrus but did not change puberty onset or GABA transmission to these cells. In contrast, activating GnRH neurons had no effect on reproductive parameters or firing rate and did not rescue reproductive phenotypes in PNA mice. These studies support the hypothesis that prepubertal neuronal activity sculpts elements of the adult reproductive neuroendocrine axis and cyclicity but indicate that other PNA-induced programming actions are required for full reproductive phenotypes and/or that compensatory mechanisms overcome activity-mediated changes to mitigate reproductive changes in adults.

## Significance Statement

Gonadotropin-releasing hormone (GnRH) neuron activity and associated GnRH release link the neuronal control of reproduction to peripheral secretion of reproductive hormones. Prenatally androgenized (PNA) mice mimic neuroendocrine aspects of polycystic ovary syndrome (PCOS). PNA reduces prepubertal GnRH neuron activity but increases adult activity. Here, we reveal that prepubertal suppression of GnRH neuron activity without androgen exposure leads to similar increases in adult GnRH neuron activity, and a mild degradation in reproductive cycles. Increasing GnRH neuron activity before puberty, however, fails to rescue cycles in PNA mice. This provides a clearer understanding of the role prepubertal GnRH neuron activity plays in establishing adult reproductive function and suggests additional androgen-dependent programming actions are required for complete reproductive disruption in this model and perhaps PCOS.

## Introduction

Reproduction is required for the perpetuation of individual species and is controlled through the hypothalamic-pituitary-gonadal axis. Gonadotropin-releasing hormone (GnRH) neurons are the final central neuronal output of this axis; they release GnRH from terminals in the median eminence to regulate luteinizing hormone (LH) and follicle-stimulating hormone (FSH) synthesis and secretion by the anterior pituitary. LH and FSH activate gonadal functions, including steroidogenesis. Gonadal-steroid feedback regulates hormone release at both the level of the brain and the pituitary. Disruptions in this axis can lead to infertility, which is estimated to affect up to one in six couples (Hartman et al., 2006; Thoma et al., 2013). The leading cause of infertility in women of childbearing age is polycystic ovary syndrome (PCOS; McCartney et al., 2002). Hyperandrogenemic PCOS affects 8–10% of women and is characterized by oligo/anovulation, mildly elevated androgens, and persistent high frequency of LH, and presumably GnRH, release (Livadas et al., 2014). Most studies of PCOS have occurred in adults, a time when reduced fertility is easily noted and diagnosis based on established criteria is possible. Increasing evidence, however, suggests aspects of PCOS emerge before and/or during the pubertal transition (McCartney et al., 2007; Burt Solorzano et al., 2010; Collins et al., 2014).

To study mechanistic underpinnings of PCOS at a neuronal level, animal models are needed. Prenatal androgenization (PNA) recapitulates many aspects of PCOS in several species including rodents, primates and sheep (Abbott et al., 1998; Foecking et al., 2005; Padmanabhan and Veiga-Lopez, 2013). Recent studies in mice demonstrated GnRH neurons are active and receive synaptic inputs well before outward signs of reproduction are present (Dulka and Moenter, 2017; Berg et al., 2018). PNA treatment altered both GnRH neuron firing rate (Roland and Moenter, 2011; Dulka and Moenter, 2017) and GABA transmission to these cells before puberty, as well as in adulthood (Sullivan and Moenter, 2004; Berg et al., 2018). Early neuronal activity in other areas of the brain contributes to organization of neuronal networks by attracting and pruning synaptic inputs (Katz and Shatz, 1996; Andreae and Burrone, 2014), but the role of prepubertal GnRH neuron activity with regard to the reproductive neuroendocrine system is not known.

The above correlational studies pose interesting questions regarding possible programming roles of PNA exposure versus changes that occur subsequent to PNA-induced alterations in neuronal activity. Specifically, PNA treatment reduces GnRH neuron activity relative to controls before puberty (Dulka and Moenter, 2017). Is it androgen exposure that leads to increased GnRH neuron activity and increased excitatory (DeFazio et al., 2002) GABAergic input in adult PNA mice, or can changing prepubertal GnRH neuron activity alone produce or rescue the reproductive phenotype? We hypothesized that decreasing GnRH neuron activity during the prepubertal period in control mice would lead to similar phenotypes observed in PNA mice including increased GnRH neuron activity and GABAergic inputs to these cells in adulthood. We also hypothesized that activating these cells before puberty in PNA mice would rescue adult reproduction. To test these postulates, we used a chemogenetic approach to modify GnRH neuron activity before puberty, and subsequently monitor adult neurophysiology and reproductive parameters.

## Materials and Methods

All chemicals were acquired from Sigma-Aldrich unless noted.

### Animals

**Figure 1.**
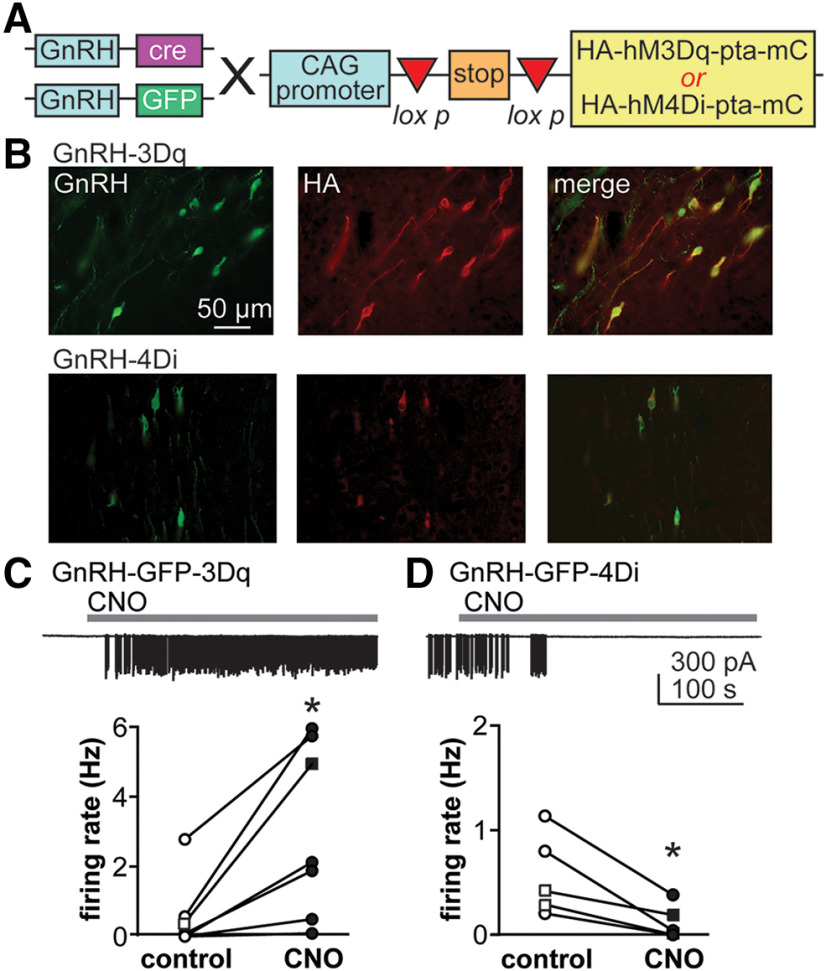
DREADD expression and function in GnRH neurons. ***A***, Mice homozygous for Cre and GFP were bred to mice expressing either the 3Dq or 4Di under the CAG promoter; mC, mCitrine. ***B***, Dual immunofluorescence for GnRH (green) and HA tag (red) in P14 female GnRH-3Dq (top) and GnRH-4Di (bottom) female mice. ***C***, ***D***, CNO alters GnRH neuron firing rate *in vitro* in GnRH-GFP-3Dq (***C***) and GnRH-GFP-4Di (***D***) mice. Scale bar is the same in ***C***, ***D***; open symbols, control; closed symbols, CNO treatment. Circles show adults, squares prepubertal mice; *p < 0.05 two-tailed Wilcoxon matched-pair signed-rank test (***C***), two-tailed paired Student’s *t* test (***D***).

Transgenic mice (C57Bl6/J) expressing Cre recombinase (Cre) under the GnRH promoter in a BAC construct (GnRH-Cre mice, JAX 021207; Yoon et al., 2005) were crossed onto mice expressing green fluorescent protein (GFP) under control of the GnRH promoter [Tg(GnRH1-EGFP)51Sumo MGI:6158457, GnRH-GFP mice, JAX 033639; Suter et al., 2000] until homozygous for both GFP and Cre (GnRH-GFP/Cre mice). Homozygous GnRH-Cre and GnRH-GFP/Cre mice were then crossed to mice expressing a floxed cassette encoding one of two versions of a designer receptor exclusively activated by designer drugs (DREADD), mCitrine and the hemagglutinin (HA) tag (Fig. 1*A*). In these mice, when Cre is present, a floxed stop cassette is removed and the CAG promoter drives expression of the cassette. The DREADDs used in these studies were hM3Dq (3Dq, JAX, 026220) and hM4Di (4Di, JAX, 026219), which activate the canonical Gq and Gi pathways, respectively. These receptors are activated by the DREADD ligand clozapine n-oxide (CNO) or its metabolite, clozapine (Gomez et al., 2017). Table 1 shows the mouse genotypes used for the experiments presented in the following studies and the abbreviations used for these in the text. All mice used in this study were heterozygous for GnRH-Cre, GnRH-GFP and CAG-DREADD (hM3Dq or hM4Di), unless noted.

**Table 1:** Genotypes of mice and in text abbreviation used

Abbreviation	Genotype
GnRH-Cre	GnRH-Cre^+/+^ or GnRH-Cre^+/-^
GnRH-GFP	GnRH-GFP^+/+^ or GnRH-GFP^+/-^
GnRH-Cre/GnRH-GFP	GnRH-Cre^+/+^/GnRH-GFP^+/+^
GnRH-4Di	GnRH-Cre^+/-^/CAG-hM4Di^+/-^
GnRH-3Dq	GnRH-Cre^+/-^/CAG-hM3Dq^+/-^
GnRH-GFP-4Di	GnRH-Cre^+/-^/GnRH-GFP^+/-^/CAG-hM4Di^+/-^
GnRH-GFP-3Dq	GnRH-Cre^+/-^/GnRH-GFP^+/-^/CAG-hM3Dq^+/-^

Because this study focused on the PNA phenotype as a model for PCOS in women, these studies only used female mice. Adult females (estrous cycles were monitored from 9 to 12 weeks of age and again two weeks before electrophysiological recordings performed at 18–43 weeks of age). All ovary-intact mice used for experiments were in the diestrous cycle stage verified via vaginal cytology. All mice had *ad libitum* access to water and chow (Teklad 2916, breeders received high protein 2919 chow, Envigo). Mice were housed on a 14/10 h light/dark cycle with time of lights on at 0300 Eastern Standard Time. Some mice were ovariectomized (OVX) for LH assay studies, and some mice received subcutaneous osmotic pumps that were removed one week later (procedure described below). Surgery was done under isoflurane anesthesia and bupivacaine applied as a local analgesic. PNA mice were generated as described (Sullivan and Moenter, 2004; Dulka and Moenter, 2017). In brief, male C57Bl/6J mice were crossed with female GnRH-GFP mice. Pregnant GnRH-GFP female mice were injected subcutaneously with 225 µg dihydrotestosterone (DHT; 5α-androstan-17β-ol-3-one) in sesame oil on days 16–18 of gestation (d1 = copulatory plug). Control groups included mice injected with sesame oil vehicle and uninjected dams. As previously reported (Roland et al., 2010; Roland and Moenter, 2011; Dulka and Moenter, 2017; Berg et al., 2018; Dulka et al., 2020), no difference (all *p* > 0.1) was observed between offspring of uninjected and vehicle-treated dams [GnRH-GFP-4Di: cycles two-way, repeated-measures ANOVA control vs vehicle *F*_(1,9)_ = 0.5455; GnRH-GFP-3Dq: vaginal opening (VO) two-way ANOVA control vs vehicle *F*_(1,32)_ = 1.802, first estrus *F*_(1,30)_ = 0.7714, extracellular recordings two-way ANOVA *F*_(1,12)_ = 3.063, cycles three-way, repeated-measures ANOVA *F*_(1,13)_ = 0.6290; all other groups had only uninjected offspring in the control groups]. Data from these groups were thus combined and reported as controls. A second dam (CD1 background strain) was included in all breeding cages for maternal and nutritional support to increase survival of PNA pups; combined litters were adjusted to <15 pups by culling CD1 pups, which are phenotypically distinct, to normalize nutrition. The Institutional Animal Care and Use Committee of the University of Michigan (PRO00006816/PRO00008797) approved all animal procedures.

### Immunohistochemistry

To assess expression of DREADD receptors in GnRH neurons, GnRH-3Dq mice and GnRH-4Di mice were perfused transcardially with 4% paraformaldehyde (Fisher) on postnatal day (P)14 or during adulthood. Brains were post fixed for 4–24 h at 4°C, then stored in 20% sucrose with 0.01% sodium azide, for at least 12 h for cryoprotection. Brains were sectioned at 30 µm into five series on a SM2010 R freezing microtome (Leica Biosystems). Sections were taken from just caudal to the olfactory bulb through the optic chiasm for use in free-floating dual immunofluorescence using standard procedures (Bellefontaine et al., 2014). The primary antibodies used (Table 2) were rat anti-HA high-affinity (1:1000, Roche) and rabbit anti-GnRH (EL-14, RRID: AB_2715535, 1:10,000; generous gift from Oline Rønnekleiv, Oregon Health and Science University, Portland, OR; Ellinwood et al., 1985). Primary antibodies were visualized with Alexa Fluor 546/594-conjugated goat anti-rat and Alexa Fluor 488-conjugated goat anti-rabbit (A-11 081 and A-11 034, Thermo Fisher Scientific), respectively. For visualization of the DREADD receptor in GnRH-4Di and GnRH-3Dq mice, sections were mounted on Superfrost Plus glass slides (Thermo Fisher Scientific) and coverslipped with ProLong Gold antifade reagent containing 4′,6-diamidino-2-phenylindole (Thermo Fisher Scientific). Immunofluorescence was detected using a fluorescent Axio Imager microscope (Zeiss). The number of cells expressing GnRH, HA, or both was counted in all sections from a single series from each mouse.

**Table 2:** Antibodies used for detection of DREADD expression in GnRH neurons

Peptide target	Antigen sequence	Name of antibody	Source, catalog number, RRID information	Species/type
Hemagglutinin (HA)	HA peptide sequence (YPYDVPDYA)	Anti-HA High Affinity	Roche catalog #11867431001, RRID:AB_390919	Rat monoclonal antibody (clone 3F10)
GnRH	GnRH conjugated to bovine serum albumin	EL-14	Dr. Oline Rønnekleiv, Oregon Health and Science University, RRID: AB_2715535	Rabbit/polyclonal

### Tail-tip blood collection and LH measurements

All mice used for blood sampling were handled for at least two weeks before experiments. Tail-tip blood collection was performed as described (Czieselsky et al., 2016). After a small nick at the tail tip, mice were placed on a flat surface and allowed to roam freely while 6 µl of tail blood was collected and immediately mixed with 54 µl of 0.1 m PBS (Invitrogen) containing 0.05% Tween 20 and 0.2% BSA (Jackson ImmunoResearch). Samples were kept on ice during blood collection then stored at −20°C until LH assay. Intraassay CV was 2.2% and interassay CVs were 7.3% [low quality control (QC), 0.13 ng/ml], 5.0% (medium QC, 0.8 ng/ml), and 6.5% (high QC, 2.3 ng/ml). Functional sensitivity was 0.016 ng/ml (Steyn et al., 2013). In some experiments, immediately following the last sample of the frequent sampling period, mice received a single injection of GnRH (150–200 ng/kg, i.p.; Bachem), and blood was collected 15 min later to test pituitary LH response.

### Control experiments to test the bioactivity of DREADDs expressed in GnRH neurons

#### Experiment 1

To test the effect of CNO on GnRH neuron firing rate, basal GnRH activity was measured for 10 min via extracellular recordings (described below) followed by bath application of CNO (0.2–1 μm) for at least 10 min in brain slices from prepubertal (P14 and P21) and adult mice. Firing rate was quantified during the final 4 min before CNO treatment and for minutes 7 through 10 inclusive during CNO treatment (*n* = 7 cells for GnRH-GFP-3Dq and *n* = 5 cells for GnRH-GFP-4Di). Data from each age group within a genotype were combined.

#### Experiment 2: hM4Di (4Di)

To study whether activation of the 4Di DREADD targeted to GnRH neurons could decrease LH *in vivo*, adult female GnRH-Cre (*n* = 4) and GnRH-4Di (*n* = 3) mice were OVX to elevate episodic LH release. Twelve to 20 d later, tail blood for LH assay was sampled at 6-min intervals for 174 min. Mice were sampled for 54 min without treatment, then received an intraperitoneal saline injection to assess the potential effects of stress attributable to injection, followed at 114 min by CNO (0.3 mg/kg or 1 mg/kg, i.p.; Enzo Life Sciences or Tocris) and then GnRH at 180 min. LH pulses were detected by a version of Cluster (Veldhuis and Johnson, 1986) running in IgorPro using cluster sizes of two points for both peak and nadir and *t* scores of 2 for detection of increases and decreases.

#### Experiment 3

CNO can be metabolized to clozapine, which can alter function of central neural systems independent of DREADD receptors (Gomez et al., 2017). To test whether clozapine alters LH release, ovary-intact GnRH-Cre mice without either DREADD (*n* = 2) were sampled at 6-min intervals for 114 min and were given 0.95 mg/kg clozapine (Tocris) intraperitoneally at 54 min. Mean LH values before and after clozapine were compared. To test whether clozapine can alter GnRH neuron firing rate, clozapine (1 μm) was bath applied during extracellular recordings of GnRH neurons in GnRH-GFP mice and firing rate assessed as in experiment 1.

#### Experiment 4: hM3Dq (3Dq)

To test if activation of 3Dq DREADD targeted to GnRH neurons could increase LH, ovary-intact GnRH-Cre and GnRH-3Dq females (*n* = 3 each) were sampled at 6-min intervals for 132 min. CNO (1 mg/kg, i.p.) was given at 54 min. To test whether changes in LH were GnRH dependent, ovary-intact GnRH-3Dq females (*n* = 2) were injected with the GnRH receptor antagonist Antide (3 mg/kg, s.c.) 1.25 h before sampling began; CNO (1 mg/kg, i.p.) was given 30 min after sampling began. To test whether elevated LH levels were sustained and whether a lower dosage of CNO (0.3 mg/kg) was able to elicit a similar rise in LH, ovary-intact GnRH-3Dq females (*n* = 2) were sampled every 6 min for 96 min then every 30 min for an additional 5 h with one additional sample at 7.5 h. CNO (0.3 mg/kg, i.p.) was injected at 30 min.

### Main experiments to test the role of manipulating GnRH neuron activity with DREADDs

The below manipulations were the same for GnRH-GFP-4Di and GnRH-GFP-3Dq mice with the exception that PNA mice were included in the latter experiments. Because PNA treatment suppresses GnRH neuron firing rate before puberty (Dulka and Moenter, 2017), no attempt was made to further suppress GnRH neuron activity in GnRH-GFP-4Di mice.

### Prepubertal CNO injections and osmopump placement

To study the role of manipulating GnRH neuron activity before puberty, GnRH-GFP-4Di or GnRH-GFP-3Dq mice received either intraperitoneal injections of CNO 12 h apart at a dosage of 0.3 mg/kg or a miniosmotic pump implant (Alzet, 1007D: flow of 0.5 µl/h for up to 7 d) beginning at two weeks of age. Osmopumps were filled with 100 µl of either 0.5 µg/µl CNO in saline with 2.57% DMSO or saline+DMSO vehicle solution. Before surgery, all pups in a litter were removed from the dams’ cage including the pups of the CD1 foster dam. Mice in which osmopumps were inserted underwent anesthesia with 2–3% isoflurane and a longitudinal incision was made on the animal’s back and the osmospump inserted subcutaneously. Immediately before insertion, the pump was immersed in sterile 0.9% saline solution to ease insertion. Bupivacaine was applied post operatively as a local analgesic and Carprofen (5 mg/kg, sc; Zoetis Petcare) was given both before surgery and 24 h later. The incision was closed with wound clips. Mice were allowed to fully recover before being placed back with other pups and subsequently the entire litter was returned to the dam. At three weeks of age, pups were weaned and either pumps were removed using the same anesthesia and analgesia protocol or injections were ceased.

### Analysis of reproductive parameters

To test whether changing postnatal firing of GnRH neurons altered reproductive parameters, day of VO was monitored upon starting CNO injections or after osmopump insertion surgery at P14; no mouse had VO during the duration of injections or before osmopump removal at P21. Upon VO, vaginal lavage was used to determine day of first estrus. Estrous cycles were assessed by daily vaginal lavage from 9 to 12 weeks of age.

### Brain slice preparation

Solutions were bubbled with 95% O_2_/5% CO_2_ throughout the duration of experiments and at least 15 min before tissue exposure to solutions. Slices were made between 8:30 A.M. and 12 P.M. The brain was rapidly removed and placed in ice-cold sucrose saline solution containing the following: 250 mm sucrose, 3.5 mm KCl, 26 mm NaHCO_3_, 10 mm D-glucose, 1.25 mm NaH_2_PO_4_, 1.2 mm MgSO_4_, and 3.8 mm MgCl_2_. Coronal (300 µm) slices were cut with a Leica VT1200S vibrating slicer (Leica Biosystems). Slices were incubated in a 1:1 mixture of sucrose saline and artificial CSF (ACSF) containing the following: 135 mm NaCl, 3.5 mm KCl, 26 mm NaHCO_3_, 10 mm D-glucose, 1.25 mm Na_2_HPO_4_, 1.2 mm MgSO_4_, and 2.5 mm CaCl_2_ (pH 7.4) for 30 min at room temperature (21–23°C) and then transferred to 100% ACSF for an additional 30 min at room temperature before recording. Recordings were performed 1–5 h after brain slice preparation; no difference in firing patterns were evident based on time after brain slice preparation.

### Extracellular recordings

Extracellular recordings were made to monitor spontaneous action potential firing (Nunemaker et al., 2003; Alcami et al., 2012; Dulka and Moenter, 2017). Slices were placed in a chamber continuously perfused with ACSF (2–3 ml/min) and heated to 30–32°C with an inline-heating unit (Warner Instruments). To identify GnRH neurons, an Olympus BX51WI microscope was used to briefly illuminate GFP-positive cells in the preoptic area at 488 nm. A Flaming/Brown P-97 puller (Sutter Instruments) was used to pull borosilicate capillary glass (type 7052, 1.65-mm outer diameter and 1.12-mm inner diameter; World Precision Instruments) into recording micropipettes with a resistance of 2–4 MΩ. Micropipettes used for recordings were filled with a HEPES-buffered solution containing the following: 150 mm NaCl, 10 mm HEPES, 10 mm D-glucose, 2.5 mm CaCl_2_, 1.3 mm MgCl_2_, and 3.5 mm KCl. All recordings were conducted on one channel of an EPC-10 dual patch clamp amplifier using Patchmaster software (HEKA Elektronik) running on a Macintosh computer. Low resistance seals (<30 MΩ) were formed between the pipette and neuron after first exposing the pipette to the slice tissue in the absence of positive pressure. Recordings were made in voltage-clamp mode with a 0-mV pipette holding potential and signals acquired at 10 kHz and filtered at 5 kHz. Recording stability and loose seal were checked every 10 min by current response to a 5-mV hyperpolarizing voltage step. Inactive cells were treated with high-potassium ACSF (20 mm K^+^). Cells that exhibited action currents in response to K^+^ were verified to be alive and recordable, and thus quiescence data were included in analysis. For cells not responding to K^+^, data analysis was truncated at the last observed action current. One-hour recordings of GFP-identified GnRH neurons were made. No more than three extracellular recordings per animal and two cells per slice were included for analysis, and at least four mice from at least three different litters were tested per group. Variation within an animal or among littermates was not less than among all animals within a group. Cell location was mapped to an atlas (Sidman et al., 1971) after recording; no differences in recording observations were attributable to location.

### Whole-cell patch-clamp

Pipettes were pulled as described above and resistance was 2–4 MΩ when filled with the following: 140 mm KCl, 10 mm HEPES, 5 mm EGTA, 0.1 mm CaCl_2_, 4 mm MgATP, and 0.4 mm NaGTP, 300 mOsm, pH 7.2 with NaOH for recording GABAergic postsynaptic currents (PSCs). Pipettes were wrapped with Parafilm (Bemis) to reduce capacitive transients; remaining transients were electronically cancelled. A liquid junction potential of −4.9 mV was corrected online during all recordings. After achieving a >1-GΩ seal and the whole-cell configuration, membrane potential was held at −65 mV. Series resistance (Rs), input resistance (Rin), and holding current (Ihold) were measured every 2–3 min using a 5-mV hyperpolarizing step from −65 mV (mean of 16 repeats, 20-ms duration, sampled at 100 kHz, and filtered at 10 kHz). Only recordings with a Rin of >400 MΩ, Ihold of −85–20 pA, stable Rs of <20 MΩ, and a stable membrane capacitance (Cm) between 8.5 and 22 pF were used for analysis. Spontaneous GABAergic PSCs (sPSCs) were monitored in voltage-clamp mode at a holding potential of −65 mV. Current was sampled at 20 kHz and filtered at 10 kHz. ACSF contained 20 μm D-APV (Tocris), and 10 μm CNQX to block ionotropic glutamate receptors. At least two 120-s recordings were made for each cell to determine sPSC frequency. To measure activity-independent miniature PSCs (mPSCs), two to three 120-s recordings were made in the presence of 0.5 μm tetrodotoxin (TTX; Tocris).

### Analysis

Action currents or PSCs were detected off-line using custom programs in Igor Pro 6.31 and 7.02 (Wavemetrics). Extracellular data were binned at 60-s intervals and were transferred to Excel (Microsoft). Mean firing rate (Hz) and PSC frequency were calculated by dividing the total number of events by the duration of recording. For PSCs, amplitude, interval, rise time, decay, and full-width half-maximum (FWHM) are also reported. Rise time was quantified from baseline to half of the maximum amplitude of the PSC. Decay time was calculated as the time between 90% and 10% of the maximum current amplitude.

### Statistics

Statistical analyses were performed using Prism 8 (GraphPad Software). Data are reported as individual values with mean ± SEM. Data distributions were tested using a Shapiro–Wilk normality test. The null hypothesis was rejected if *p* < 0.05, and exact *p* values <0.1 are reported. Specific tests were selected based on experimental design and data distribution and are indicated in the results.

## Results

### DREADD receptors are effectively targeted to GnRH neurons

To verify DREADD expression was present in GnRH neurons at the same prepubertal age that CNO was administered in the present studies, dual immunofluorescence for GnRH peptide and the HA-tag in the DREADD transgene was performed in GnRH-3Dq and GnRH-4Di mice without GFP at P14 (Fig. 1*B*). HA expression was detected in 95% of GnRH-positive cells in P14 GnRH-3Dq animals (*n* = 2, 139/146 and 141/149 cells) and 94% of GnRH-positive cells in P14 GnRH-4Di animals (*n* = 2, 158/162 and 97/104 cells). These results indicate the majority of GnRH neurons express the DREADD receptor as expected. DREADD expression was also observed in non-GnRH neurons (GnRH-3Dq, 183 and 186 cells; GnRH-4Di, 448 and 117 cells). These cells were in the lateral septum; this was expected as GnRH is expressed in this region during development (Skynner et al., 1999), permitting Cre-lox excision of the stop cassette. HA-immunoreactivity in GnRH neurons extended into processes (Fig. 1*B*), as well as the median eminence (data not shown).

### Results of control experiments to test the bioactivity of DREADDs expressed in GnRH neurons

#### Experiment 1

The efficacy of activating DREADDs with the CNO ligand was first tested *in vitro* in brain slices. In brain slices from GnRH-GFP-3Dq mice, CNO (0.2-1 μm) increased firing rate of GnRH neurons (*n* = 7, control 0.6 ± 0.4 Hz, CNO 3.0 ± 0.9 Hz, *p* = 0.0156 two-tailed Wilcoxon matched-pairs signed-rank test, W = 28; Fig. 1*C*). In brain slices from GnRH-GFP-4Di mice, CNO (0.2 μm) decreased firing rate of GnRH neurons (*n* = 5, control 0.6 ± 0.2 Hz, CNO 0.1 ± 0.1 Hz, *p* = 0.0266, two-tailed paired Student’s *t* test, *t* = 3.426, df = 4; Fig. 1*D*).

#### Experiment 2: CNO decreases LH pulses in OVX GnRH-4Di mice

**Figure 2.**
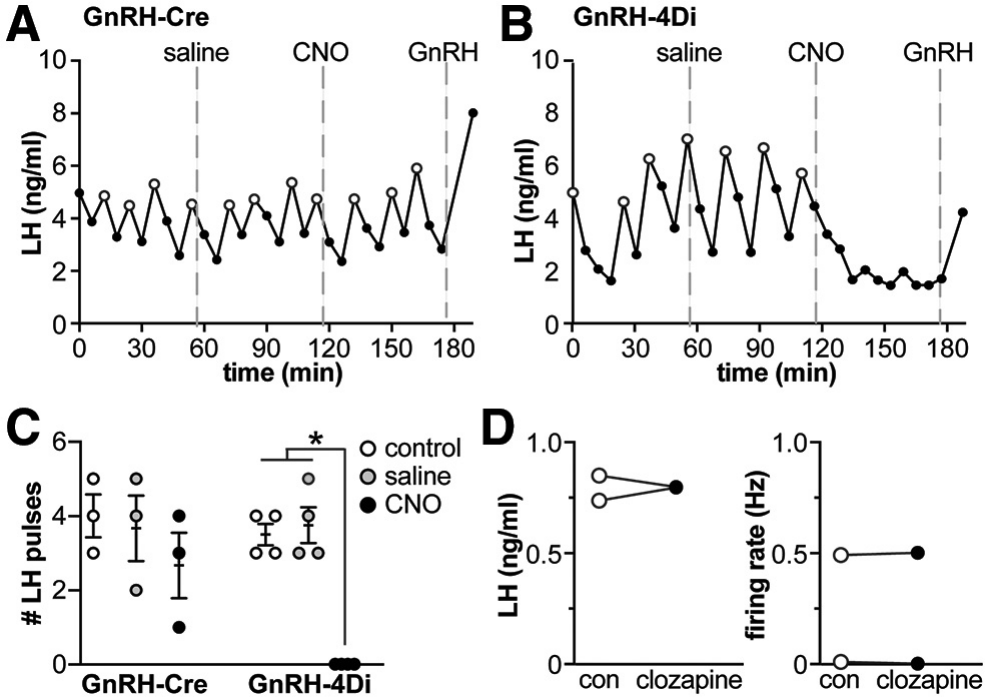
Activation of 4Di-coupled DREADDs targeted to GnRH neurons reduces LH. ***A***, ***B***, LH pulse patterns in OVX GnRH-Cre mice (***A***) or OVX GnRH-4Di mice (***B***) during a control period, after intraperitoneal saline injection, after intraperitoneal CNO (1 mg/kg) injection, and after intraperitoneal GnRH (150–200 ng/kg) injection. Cluster detected pulses before GnRH treatment are shown as white symbols, dashed gray lines show times of intraperitoneal injections. ***C***, Individual values and mean ± SEM number of LH pulses during the three treatment periods in ***A***, ***B***, two-way repeated-measures ANOVA (interaction *F*_(2,10)_ = 6.820, *p* = 0.0135; genotype *F*_(1,5)_ = 2.770, *p* = 0.1569; treatment *F*_(2,10)_ = 24.86, *p* = 0.0001; subject *F*_(5,10)_ = 3.716, *p* = 0.0368, Sidak *post hoc*; *p < 0.0001). ***D***, left, Mean LH values in samples before and after intraperitoneal injection of 0.95 mg/ml clozapine to GnRH-Cre mice. Right, Mean GnRH neuron firing rate for 4 min before and minutes 7–10 during 1 μm clozapine treatment.

Baseline LH levels in naturally cycling mice are suppressed by steroidal feedback; therefore, effectiveness of the 4Di DREADD was assessed in OVX mice, which have elevated LH due to release from negative feedback. In GnRH-Cre mice lacking 4Di, neither saline nor CNO (0.3 mg/kg) altered LH pulses (*n* = 3, two-way repeated-measures ANOVA/Sidak; for statistical parameters, see legend to Fig. 2*A*,*C*). In contrast, in GnRH-4Di mice, saline had no effect while CNO abolished LH pulses (*n* = 4, *p* < 0.0001 vs control and saline within subject, two-way repeated-measures ANOVA/Sidak; Fig. 2*B*,*C*).

#### Experiment 3: clozapine does not alter LH release or GnRH neuron firing rate in non-DREADD expressing mice

Studies have suggested that CNO activation of DREADDs may occur via clozapine, a metabolic product of CNO, and that some actions thought to be CNO-mediated may actually be effects of clozapine (Gomez et al., 2017). To determine whether clozapine alters LH levels independent of DREADD expression, we injected ovary-intact GnRH-Cre mice lacking DREADDS with clozapine (0.95 mg/kg, i.p.). Clozapine had no effect on mean LH levels *in vivo* or on firing rate of GnRH neurons *in vitro* (*n* = 2 each; Fig. 2*D*). This, and the lack of effect of CNO in mice lacking DREADDs, indicates changes in LH are likely attributable to CNO activation of DREADD receptors expressed in GnRH neurons.

#### Experiment 4: CNO increases LH release in GnRH-3Dq mice

**Figure 3.**
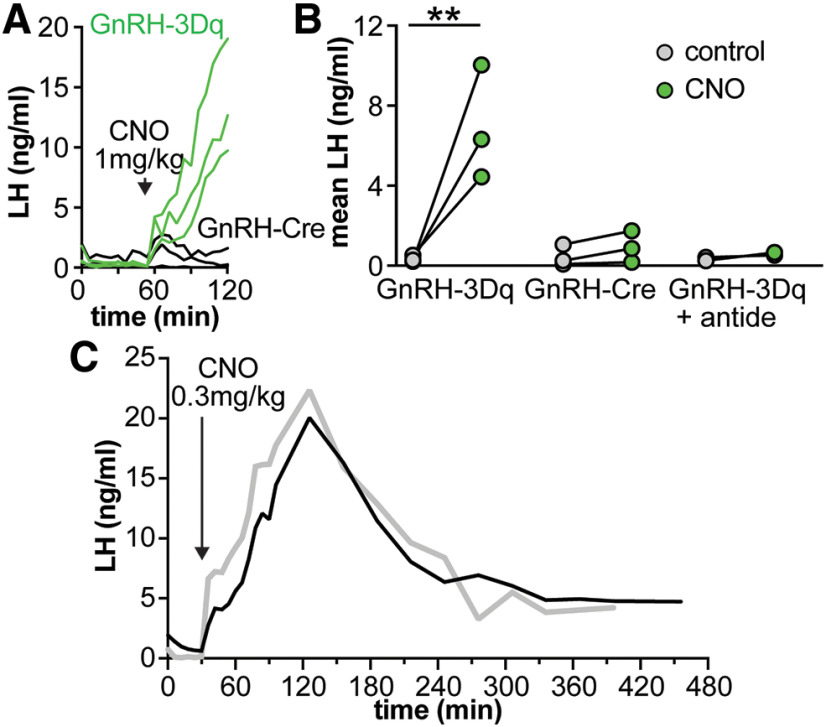
CNO induces LH release in GnRH-3Dq mice in a GnRH-dependent manner. ***A***, LH profiles in three GnRH-Cre (black) and three GnRH-3Dq mice (green) before and after intraperitoneal CNO injection (1 mg/kg; arrow). ***B***, Mean LH in 10 samples before (control) and after CNO injection (left and center) and six samples before and after CNO (right). Two-way repeated-measures ANOVA (interaction *F*_(2,5)_ = 10, *p* = 0.0174; genotype *F*_(2,5)_ = 9.6, *p* = 0.0192; treatment *F*_(1,5)_ = 13, *p* = 0.0165; subject *F*_(5,5)_ = 1, *p* = 0.5005, Sidak *post hoc*; **p < 0.01). ***C***, LH response to CNO injection (0.3 mg/kg) remains elevated for several hours after a single injection; black and gray traces are from two separate animals tested.

To test whether activation of the 3Dq DREADD in GnRH neurons increased LH release, LH pulse sampling was conducted in GnRH-Cre and GnRH-3Dq mice which were not OVX. In GnRH-Cre mice lacking 3Dq, LH levels remained near baseline after CNO (1 mg/kg) injection (*n* = 3, *p* = 0.97, two-way repeated-measures ANOVA/Sidak; for statistical parameters, see legend to Fig. 3*A*,*B*). In GnRH-3Dq mice, in contrast, LH levels rose within 6 min of the injection and remained elevated for the remainder of the sampling period (78 min; *n* = 3, *p* < 0.01 two-way ANOVA/Sidak; Fig. 3*A*). The CNO-elicited increase was GnRH dependent, as pretreatment of GnRH-3Dq mice with the GnRH receptor antagonist Antide (3 mg/kg) blocked the CNO-elicited LH increase (*n* = 2, *p* = 0.99, two-way ANOVA/Sidak; Fig. 3*B*). Subsequent studies revealed the increase induced by a single CNO injection was maintained for several hours (*n* = 2; Fig. 3*C*) and could be elicited by a lower dose (0.3 mg/kg) of CNO. Thus, CNO effectively activates the reproductive neuroendocrine axis in this animal model and can do so for a prolonged period. The lower dose of 0.3 mg/kg was used for subsequent experiments to manipulate firing activity in prepubertal mice.

### Results of main experiments to test the role of manipulating GnRH neuron activity with DREADDs

**Figure 4.**
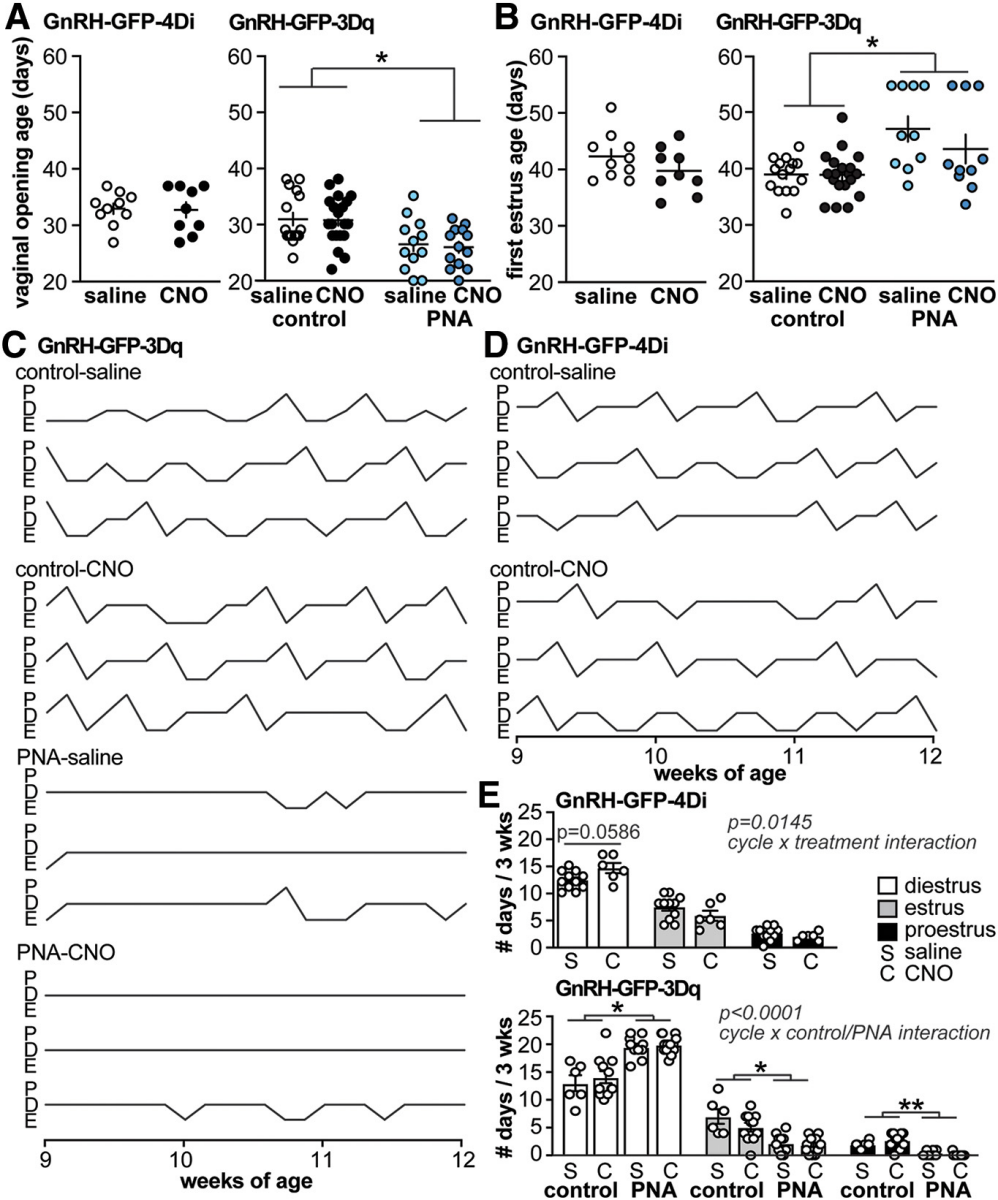
Reproductive parameters are not altered by prepubertal administration of CNO. ***A***, ***B***, Age at VO (***A***) and first estrus (***B***) in GnRH-GFP-4Di (left) and GnRH-GFP-3Dq (right) mice; *p < 0.05 two-way ANOVA/Sidak. ***C***, ***D***, Representative cycles (P, proestrus; D, diestrus; E, estrus) from GnRH-GFP-3Dq (***C***) and GnRH-GFP-4Di (***D***) mice. ***E***, Individual values and mean ± SEM of number of days spent in each cycle stage; *p < 0.05, **p < 0.01 three-way repeated-measures ANOVA/Fisher.

Puberty measures were not affected by altering GnRH neuron activity during prepubertal development. To assess whether changing GnRH neuron activity during prepubertal development altered reproduction, three parameters were examined: timing of VO (the first outward sign of puberty), timing of first estrus, and reproductive cycles in adults. CNO was administered from two to three weeks of age via either intraperitoneal injection every 12 h or osmopump. No difference was observed between treatment methods (two-tailed unpaired Mann–Whitney *U* or Student’s *t* test as appropriate; data not shown), and these data are combined. Because osmopumps were judged to be less stressful on the mice, some groups were done entirely with osmopumps (i.e., firing rate of GnRH-GFP-3Dq PNA saline and GABAergic PSC recordings in GnRH-GFP-4Di mice). No differences were observed between saline and CNO treatment in GnRH-GFP-4Di or GnRH-GFP-3Dq control mice in either the age at VO or age at first estrus (Fig. 4*A*,*B*, 4Di left panel, 3Dq right panel; Table 3). Despite no difference between saline-treated and CNO-treated GnRH-GFP-3Dq control groups, the expected effects of PNA treatment on VO and first estrus were observed. Specifically, PNA treatment advanced VO and delayed first estrus, regardless of saline or CNO treatment.

**Table 3:** Statistical parameters for reproductive measures

	*n*	Results
GnRH-GFP-4Di (two-tailed unpairedStudent’s *t* test)		
VO (4*A*, left panel)	10 saline 9 CNO	*p* = 0.8931, *t* = 0.1365, df = 17
Age at first estrus (4*B*, left panel)	10 saline 9 CNO	*p* = 0.1936, *t* = 0.1353, df = 17
GnRH-GFP-3Dq (two-way ANOVA)		
VO (4*A*, right panel)	16 control saline 20 control CNO 12 PNA saline 13 PNA CNO	*Control vs PNA: *F*_(1,57)_ = 17.11, *p* = 0.0001 Saline vs CNO: *F*_(1,57)_ = 0.0897, *p* = 0.766 Interaction: *F*_(1,57)_ = 0.0204, *p* = 0.887
Age at first estrus (4*B*, right panel)	15 control saline 19 control CNO 10 PNA saline 10 PNA CNO	*Control vs PNA: *F*_(1,50)_ = 18.45, *p* < 0.0001 Saline vs CNO: *F*_(1,50)_ = 1.349, *p* = 0.2510 Interaction: *F*_(1,50)_ = 1.12, *p* = 0.2950
Estrous cycles		
GnRH-GFP-4Di (two-way ANOVA)	11 saline 6 CNO	Saline vs CNO: *F*_(1,45)_ = 0, *p* > 0.9999 *Cycle stage: *F*_(2,45)_ = 151.1, *p* < 0.0001 *Interaction: *F*_(2,45)_ = 4.654, *p* = 0.0145
GnRH-GFP-3Dq (three-way ANOVA)	6 control saline 11 control CNO 10 PNA saline 14 PNA CNO	*Cycle stage: *F*_(1.084,40.10)_ = 257.8, *p* < 0.0001 *Control vs PNA: *F*_(1,37)_ = 0.0178, *p* = 0.895 Saline vs CNO: *F*_(1,37)_ = 1.237, *p* = 0.2733 *Cycle stage × control vs PNA: *F*_(2,74)_ = 20.0, *p* < 0.0001 Cycle stage × saline vs CNO: *F*_(2,74)_ = 1.085, *p* = 0.3423 Control vs PNA × saline vs CNO: *F*_(1,37)_ = 0.0178, *p* = 0.895 Cycle stage × control vs PNA × saline vs CNO: *F*_(2,74)_ = 0.477, *p* = 0.622

### Estrous cycles were mildly altered by inhibiting GnRH neuron activity during prepubertal development

Estrous cycles were assessed from 9 to 12 weeks of age to determine whether they were altered by modulation of prepubertal GnRH neuron activity. Representative cycles from each group are displayed in Figure 4*C*,*D*, quantification of days spent in each cycle stage are shown in Figure 4*E*, and statistical comparisons are in Table 3. In GnRH-GFP-4Di mice, there was an interaction between cycle stage and treatment (i.e., saline vs CNO), attributable primarily by a mild increase in days spent in diestrus in mice that had been treated with CNO during the prepubertal period. In GnRH-GFP-3Dq mice, there was no effect of saline versus CNO treatment, but the expected difference between cycle stage and animal model (i.e., control vs PNA) was observed (Sullivan and Moenter, 2004; Dulka and Moenter, 2017). Specifically, cyclicity was disrupted in PNA mice when compared with control animals, with more days in diestrus and fewer days in both estrus and proestrus (three-way ANOVA; Fig. 4*E*, bottom; Table 3).

### Prepubertal reduction of GnRH neuron activity increases GnRH neuron firing rate in adults but does not alter GABAergic transmission to these cells

**Figure 5.**
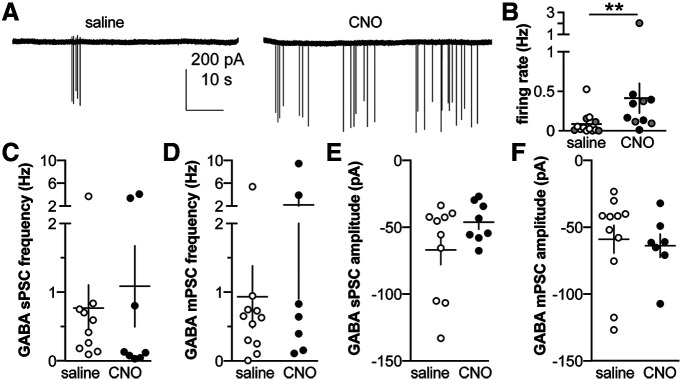
Reducing GnRH neuron activity in GnRH-GFP-4Di mice from two to three weeks of age changes GnRH neuron firing rate but not GABAergic transmission in adults. ***A***, Representative extracellular recordings from each group. ***B–F***, Individual values and mean ± SEM of GnRH neuron firing rate (***B***), GABA sPSC frequency (***C***), GABA mPSC frequency (***D***), GABA sPSC amplitude (***E***), and GABA mPSC amplitude (***F***) in GnRH-GFP-4Di mice; **p < 0.01 two-tailed Mann–Whitney *U* test. Gray symbols in ***B*** indicate mice receiving twice daily intraperitoneal injections of saline or CNO, open and black symbols denote osmopump administration of saline and CNO, respectively. Note that elimination of the cell firing at >2 Hz in ***B*** does not alter the observation of a significant difference in firing rate.

To assess whether decreasing GnRH neuron firing during the prepubertal period alters adult GnRH neuron firing and/or GABAergic transmission to GnRH neurons, electrophysiological recordings were made in brain slices from GnRH-GFP-4Di control mice. Representative recordings from each group are shown in Figure 5*A*. CNO treatment from two to three weeks of age increased firing rate of GnRH neurons from these mice in adulthood (Fig. 5*B*; Table 4). This result is similar to what has been observed in PNA mice in which the decreased GnRH neuron activity observed before puberty is correlated with elevated GnRH neuron activity assessed in adulthood (Roland and Moenter, 2011; Dulka and Moenter, 2017). We therefore tested whether GABAergic transmission was also elevated in adulthood following prepubertal suppression of GnRH neurons as is observed in adult PNA mice (Sullivan and Moenter, 2004; Berg et al., 2018). There was no change in frequency or amplitude of spontaneous or miniature GABAergic PSCs to GnRH neurons in adults attributable to prepubertal treatment (Fig. 5*C–F*; Table 4). There were no differences in passive properties between groups (Table 5).

**Table 4:** Statistical parameters for comparisons of firing rate and GABAergic PSCs in GnRH-GFP-4Di mice (two-tailed Mann–Whitney *U* test)

Comparison	*n* cells	*U*	*p* value
Extracellular recordings			
Frequency (Hz)	12 saline, 9 CNO	16	*p* = 0.0056
Spontaneous GABAergic PSCs			
Frequency (Hz)	10 saline, 8 CNO	29	*p* = 0.3599
Amplitude (pA)	10 saline, 8 CNO	39	*p* = 0.3599
Miniature GABAergic PSCs			
Frequency (Hz)	11 saline, 7 CNO	33	*p* = 0.6590
Amplitude (pA)	11 saline, 7 CNO	28	*p* = 0.3749

**Table 5:** Passive property values and comparisons for GABAergic sPSC and mPSC recordings in GnRH-GFP-4Di mice (mean ± SEM, *p* values from two-tailed unpaired Student’s *t* test with or without Welch correction)

	Saline	CNO	*p* value^*^	*t*	df
Spontaneous GABAergic PSCs					
*n*	10	8			
Series resistance (MΩ)	12.8 ± 0.9	11.4 ± 0.7	0.260	1.167	16
Input resistance (MΩ)	602.9 ± 41.6	660.4.6 ± 53.6	0.402	0.861	16
Holding current (pA)	–54.0 ± 7.3	–49.0 ± 6.9	0.629	0.492	16
Capacitance (pF)	14.4 ± 1.2	13.9 ± 0.5	0.710	0.380	12.4
Miniature GABAergic PSCs					
*n*	11	7			
Series resistance (MΩ)	13.2 ± 1.1	12.6 ± 1.3	0.760	0.310	16
Input resistance (MΩ)	681.0 ± 62.4	734.4.4 ± 116.9	0.665	0.441	16
Holding current (pA)	–49.4 ± 4.3	–53.8 ± 10.5	0.660	0.440	16
Capacitance (pF)	13 ± 1.1	14.7 ± 1.1	0.609	0.522	16

* compares listed passive properties between saline and CNO groups.

### Prepubertal increase of GnRH neuron activity does not alter firing rate in adults

**Figure 6.**
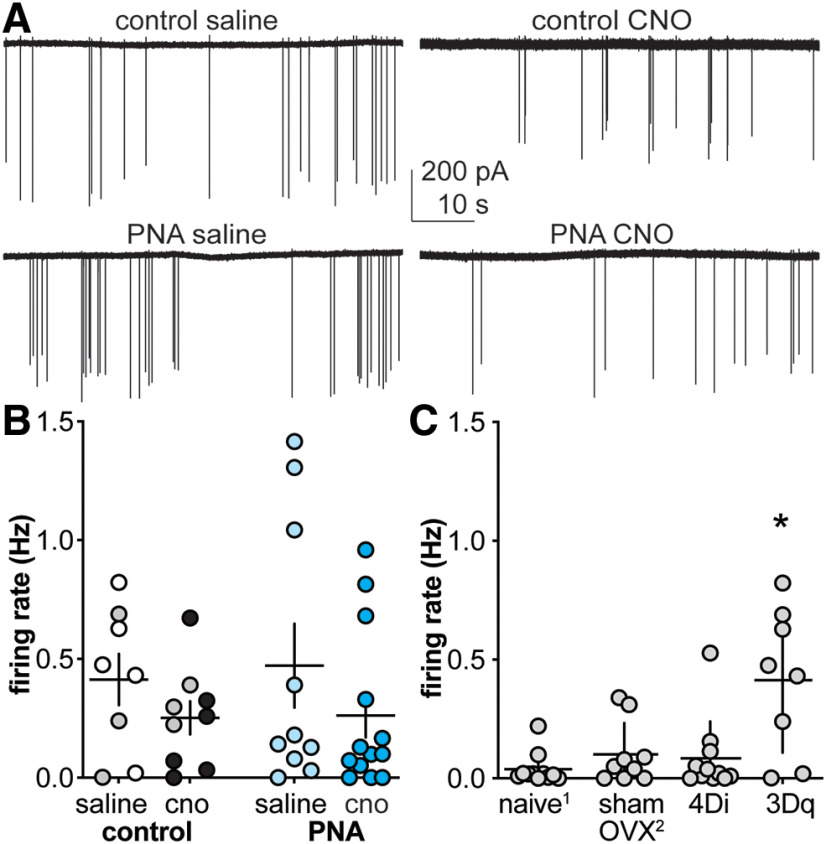
Prepubertal increase of GnRH neuron activity in GnRH-GFP-3Dq mice does not alter adult GnRH neuron activity when compared with controls, but expression of the 3Dq receptor increases control GnRH neuron activity. ***A***, Representative extracellular recordings from each group. ***B***, Individual values and mean ± SEM GnRH neuron firing rate for GnRH-GFP-3Dq mice. Gray symbols in ***B*** indicate mice receiving twice daily intraperitoneal injections of saline or CNO, open symbols denote osmopump administration of saline and black symbols osmopump administration of CNO; all PNA mice received osmopumps. ***C***, Individual values and mean ± SEM comparison of firing rate from diestrous vehicle controls over three different experiments examining firing rate versus that in PNA mice. ^1^Naive animals were unmanipulated (data from Dulka and Moenter, 2017); ^2^sham animals received sham OVX surgery (data from Dulka et al., 2020). ^*^Kruskal–Wallis (KW = 8.361, *p* = 0.0391)/Dunn’s versus naive *p* = 0.0169.

To assess whether increasing GnRH neuron activity during the prepubertal period in PNA mice could normalize firing rate in adulthood, electrophysiological recordings were made in acute brain slices from GnRH-GFP-3Dq PNA mice that were given saline or CNO from two to three weeks of age. Representative recordings from each group are shown in Figure 6*A*. No differences were observed among groups (Fig. 6*B*; Table 6). Of note, the increased firing rate repeatedly observed in PNA relative to control diestrous mice was not observed (Roland and Moenter, 2011; Dulka and Moenter, 2017; Dulka et al., 2020). Firing rate in cells from saline-treated GnRH-GFP-3Dq control mice appeared elevated. To test this, we compared the firing rate of GnRH neurons in these mice to the saline-treated GnRH-GFP-4Di mice from this study and diestrous control groups from two prior studies in which animals were naive (unmanipulated; Dulka and Moenter, 2017) or sham OVX (Dulka et al., 2020). This comparison revealed the firing rate of cells from GnRH-GFP-3Dq controls was indeed elevated (Fig. 6*C*; Table 6). This observation suggests ligand-independent activation of 3Dq may occur. Because of this caveat, studies on GABA transmission were not conducted in mice with the 3Dq DREADD targeted to GnRH neurons.

**Table 6:** Two-way ANOVA and Kruskal–Wallis parameters for extracellular recordings in GnRH-GFP-3Dq and GnRH-GFP mice

Comparison (figure)	Control vs PNA	Saline vs CNO	Interaction
Two-way ANOVA for firing rate (Hz) inGnRH-GFP-3Dq mice (6*A*)	*F*_(1,35)_ = 0.021, *p* = 0.885	*F*_(1,35)_ = 1.865, *p* = 0.181	*F*_(1,35)_ = 0.104, *p* = 0.749
	Naive vs sham	Naive vs 4Di	Naive vs 3Dq
Kruskal–Wallis (KW = 8.361)/Dunn (6*B*)	*p* > 0.99	*p* > 0.99	*p* = 0.0169

## Discussion

In PNA mice, neurobiological changes before puberty are correlated with alterations in both neurobiology and reproductive function in adulthood. Prior work could not separate the effects of PNA treatment from the effects of subsequent changes in GnRH neuron activity, specifically reduced GnRH neuron firing rate before puberty, on these adult outcomes. Here, we used chemogenetics to study the effects of altering prepubertal GnRH neuron activity in the absence of PNA exposure on later reproductive function. We found that decreasing prepubertal GnRH neuron activity is sufficient to reproduce PNA-induced increases in GnRH neuron firing rate and shift reproductive cyclicity toward that observed in PNA mice, but not to alter puberty timing or GABAergic transmission in adulthood. In contrast, increasing GnRH neuron activity during the prepubertal period could not rescue the negative effects of PNA exposure on the reproductive parameters that were examined.

To our knowledge, this is the first study using DREADD expression in GnRH neurons to modulate their activity. Changes in LH release *in vivo* and GnRH neuron firing in brain slices were as expected for the respective DREADD used. The present work indicates that changing prepubertal activity of GnRH neurons was indeed able to alter their adult function. Specifically, inhibition of GnRH neuron activity during postnatal/prepubertal development both increased firing rate of these cells in adults and shifted reproductive cycles toward more days in diestrus. Both of these findings are consistent with observations made in the PNA model in which decreased GnRH neuron firing rate before puberty is correlated with increased firing rate and disrupted cycles characterized by extended diestrus in adulthood (Dulka and Moenter, 2017). The present findings extend that observation by demonstrating at least partial causation between the early and later changes in neuronal activity. Similar relationships have been demonstrated elsewhere in the central nervous system, for example, neuronal activity during a critical period of postnatal development is important for proper synaptic innervation of the primary visual cortex and normal adult function in the visual system (Chattopadhyaya et al., 2004).

In PNA mice, suppressed firing rate before puberty is also correlated with increased GABAergic transmission to these cells in adulthood (Sullivan and Moenter, 2004; Berg et al., 2018). No change, however, was observed in spontaneous or miniature GABAergic PSC frequency or amplitude in GnRH neurons in mice in which prepubertal GnRH neuron activity was suppressed in the absence of PNA exposure. This suggests increased GABAergic input in PNA mice is at least in part attributable to other androgen-induced programming actions. GnRH neurons are thought to be unable to respond directly to androgens (Herbison et al., 1996). In this regard, arcuate kisspeptin neurons, which are directly androgen responsive (Smith et al., 2005), may play a critical role in generating the input to GnRH neurons needed to regulate cyclicity (Clarkson et al., 2017; Vanacker et al., 2017). Supporting this, chronic chemogenetic activation of arcuate GABAergic neurons resulted in increased LH secretion and impaired cyclicity (Silva et al., 2019). Androgens can also increase spine density on CA1 hippocampal neurons in adult male and female rats (Leranth et al., 2004; Cunningham et al., 2007). Anatomic and physiologic evidence suggests synaptic sites on GnRH neurons are increased in PNA mice (Sullivan and Moenter, 2004; Moore et al., 2015; Berg et al., 2018). Together, these observations raise the possibility that androgens play a stronger role in programing inputs to GnRH neurons than does androgen-induced suppression of GnRH neuron activity before puberty. In mice in which 4Di was used to suppress prepubertal GnRH activity, these cells may undergo homeostatic compensation of intrinsic properties to maintain GnRH neuron activity at the higher level observed in the absence of increased excitatory GABA drive (DeFazio et al., 2002; Herbison and Moenter, 2011). GABA is the primary fast synaptic input to GnRH neurons, but changes in glutamatergic inputs and/or those of peptidergic neurons in driving the increased GnRH neuron activity in these mice cannot be excluded.

In contrast to the partial phenocopy of the PNA treatment achieved by chemogenetically suppressing GnRH neurons from two to three weeks of age, chemogenetic manipulation of these cells at this time had no effect on adult neuronal firing rate or reproductive cycles. The activating experiments with the 3Dq DREADD were conducted in PNA mice to attempt to “rescue” the reproductive aspects observed in PNA adults by restoring high levels of prepubertal GnRH neuron activity. These data must be interpreted with caution as GnRH neuron firing rate was elevated in mice with 3Dq DREADD targeted to these cells even in control mice treated with saline when compared with both the 4Di DREADD cells in the present work and controls from prior studies; notably the presence of the 4Di DREADD did not appear to suppress GnRH neuron activity in adult controls (Dulka and Moenter, 2017; Dulka et al., 2020).

Neither chemogenetic activation nor suppression of GnRH neurons from two to three weeks of age altered external markers of puberty that were assessed: the timing of VO and first estrus. It is possible that the chemogenetic treatment was too proximal to VO to alter its timing. PNA treatment reduces GnRH neuron activity at this same time period and advances VO, but these activity changes are subsequent to androgen treatment (Dulka and Moenter, 2017). Together, these observations suggest that changing GnRH neuron activity alone is not sufficient to alter pubertal markers and that prenatal programming actions of androgens are needed.

There are several cautions to bear in mind when interpreting these results. First, we have no measure of how the degree of suppression induced by 4Di or activation induced by 3Dq compare to activity levels typically achieved by GnRH neurons during development *in vivo*. Failure of complete phenocopy in the 4Di or to observe any effects in the 3Dq mice may be attributable to either overshooting or undershooting the typical physiologic range. Of note, our *in vitro* controls do indicate these chemogenetic tools induce the expected direction of response. Second, the manipulations may have been done at the wrong time during development to result in later physiologic changes. In this regard, the present treatment period was chosen based on when PNA-induced alterations in GnRH neuron firing rate occur, and is at an age when activity-dependent synaptic changes have been shown to be important in other systems (Soto et al., 2012; Weinhard et al., 2018). Third, while the GnRH-Cre effectively targeted expression of both DREADDs to GnRH neurons, with >95% of these cells expressing these receptors, DREADDs were also expressed in non-GnRH neurons, particularly in the lateral septum. We thus cannot exclude possible off-target effects from CNO action via these neurons. Fourth, ligand-independent DREADD activation may occur. This has been reported for 4Di (Saloman et al., 2016) and may account for the high firing rate observed in 3Dq adult controls in the present work. Fifth, it is possible that compensatory mechanisms overcome changes induced by developmental changes in GnRH neuron activity. Finally, there may be a wide range of GnRH neuron firing rates that can support fairly regular cycles in the absence of other androgen-induced changes.

The present findings suggest that PNA-induced suppression of prepubertal GnRH neuron activity contributes to increased firing rate of these cells in adulthood but that the suppression of activity during development alone is not sufficient to phenocopy the model. This suggests that androgen exposure plays a necessary role in programming reproductive neuroendocrine changes in both the PNA model and, perhaps, in PCOS. This postulate is supported by the observation that increasing androgens in a variety of models used to mimic PCOS produce similar reproductive neuroendocrine outcomes (Dumesic et al., 1997; Foecking et al., 2005; Kauffman et al., 2015; Tata et al., 2018).

## Synthesis

Reviewing Editor: Cheryl Sisk, Michigan State University Decisions are customarily a result of the Reviewing Editor and the peer reviewers coming together and discussing their recommendations until a consensus is reached. When revisions are invited, a fact-based synthesis statement explaining their decision and outlining what is needed to prepare a revision will be listed below. Note: If this manuscript was transferred from JNeurosci and a decision was made to accept the manuscript without peer review, a brief statement to this effect will instead be what is listed below.

Authors have adequately addressed the JN reviewers’ concerns.
